# Pulmonary embolism as the initial manifestation of right atrial myxoma: A case report and review of the literature

**DOI:** 10.1097/MD.0000000000018386

**Published:** 2019-12-20

**Authors:** Guofeng Ma, Dan Wang, Yongtao He, Ruifeng Zhang, Yong Zhou, Kejing Ying

**Affiliations:** a Department of Respiratory Diseases; b Department of Radiology; c Department of Pathology, Sir Run Run Shaw Hospital, Zhejiang University School of Medicine, Hangzhou, China.

**Keywords:** atrial myxoma, diagnosis, pulmonary embolism, treatment

## Abstract

**Rationale:**

Pulmonary embolisms (PEs) are caused by emboli, which mostly originate from deep venous thrombi that travel to and suddenly block the pulmonary arteries. The emboli are usually thrombi, and right atrial myxoma emboli are rare.

**Patient concerns:**

A 55-year-old man presented with shortness of breath and syncope. We proceeded with computed tomography pulmonary angiography (CTPA) and transthoracic echocardiogram (TTE), the results of which suggested that the diagnosis was a right atrial mass.

**Diagnosis:**

A definitive diagnosis compatible with a right atrial myxoma (RAM) with tumoral pulmonary emboli after surgical excision was made.

**Intervention:**

Right atrial and pulmonary artery embolectomy.

**Outcomes:**

The patient followed an uneventful course during the 6 years of follow-up after surgery. According to a review of the literature, RAMs are often not diagnosed in a timely manner or even go completely undiagnosed. TTE, transesophageal echocardiography (TEE), CT, magnetic resonance imaging (MRI), and positron emission tomography/computed tomography may be helpful in the preoperative diagnosis. Surgical removal of the masses from the atrium and pulmonary arteries was relatively uneventful.

**Lessons:**

RAMs should be considered unlikely reasons for fatal pulmonary embolisms.

## 1 Introduction

Pulmonary embolisms (PEs) range from asymptomatic, incidentally discovered emboli to massive thromboembolisms that cause immediate death. PEs are life-threatening and have a high morbidity rate. Annually, as many as 300,000 people in the United States die from acute PEs, and in China, PEs are currently much more common than they were 10 years ago.^[1]^ Approximately 50% to 70% of the emboli of pulmonary embolisms originate from deep venous thrombosis (DVT), and most of these occur in the lower extremities. The patients without DVT should be screened for occult cancer. Although cancer-associated venous thrombosis has been widely described, emboli from benign tumors are less frequently mentioned.^[2]^ Most atrial myxoma-complicated pulmonary emboli are tumoral, and thrombotic emboli have been less frequently reported.^[3,4]^ We report a rare case of a right atrial myxoma with a pulmonary localization that mimicked a pulmonary embolism.

## 2 Case presentation

**Figure 1:**
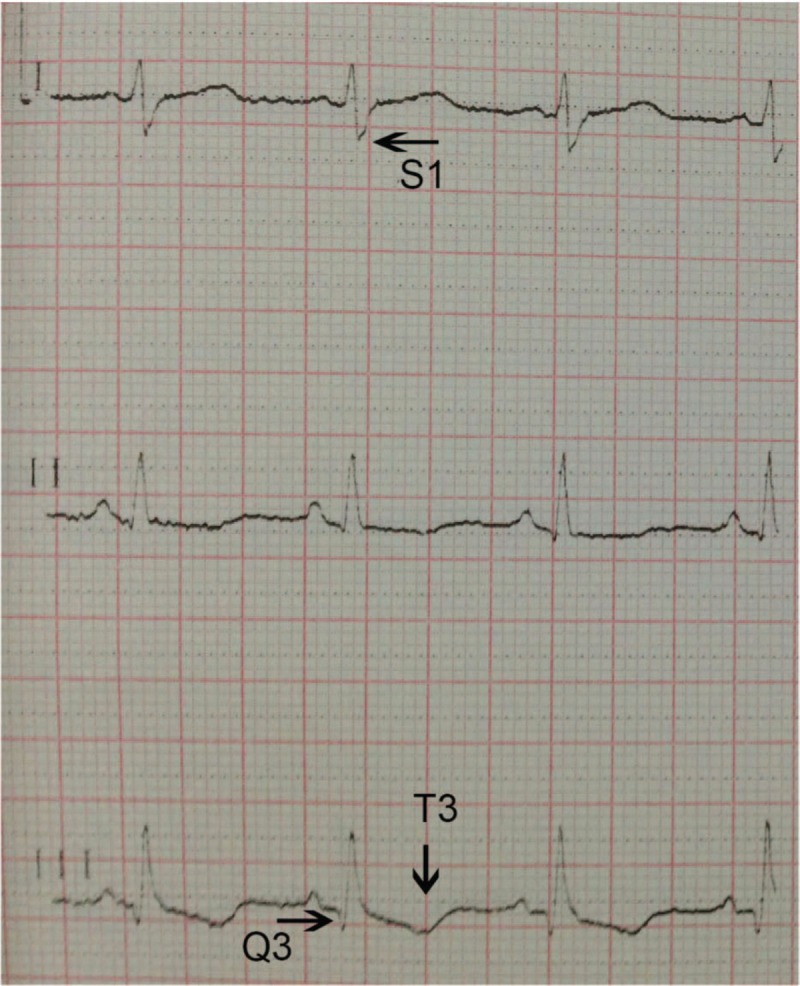
Electrocardiogram showed a S1Q3T3 pattern.

**Figure 2:**
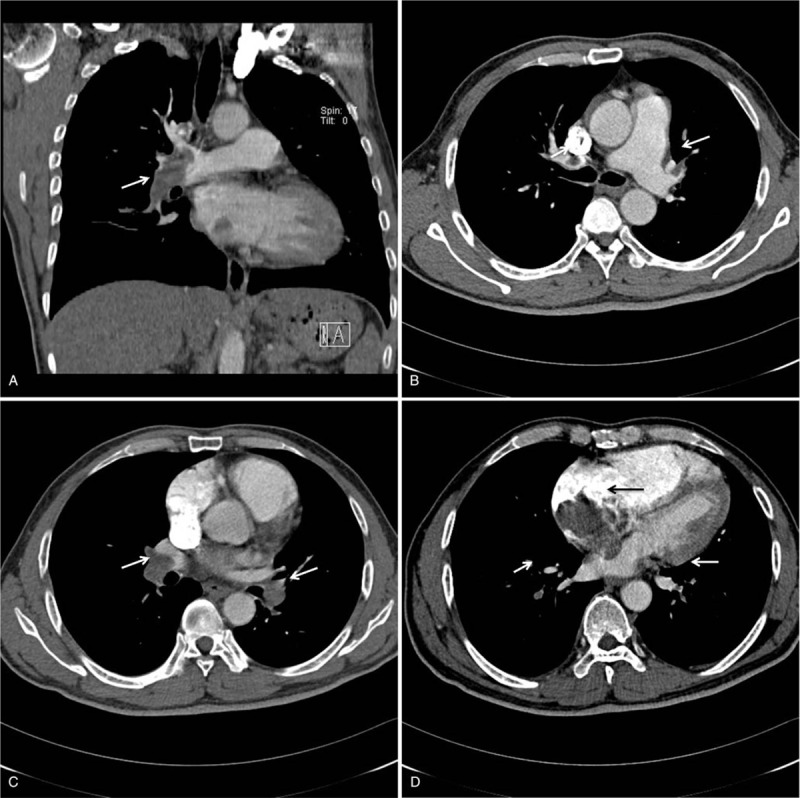
A. The coronal image shows the filling defects in the right main and lower lobe PA (white arrow). B. Transverse images show filling defects in both upper lobe PAs (white arrow). C. Transverse images showed filling defects in both lower lobe PAs (white arrow). D. The transverse image showed an irregular mass in the right atrium (black arrow) and filling defects in both lower segmental PAs (white arrow). PA = pulmonary arteries.

A 55-year-old man with no underlying diseases was admitted to the emergency room with gradually aggravated shortness of breath for 2 months and syncope and right chest pain for 6 hours. He had a habit of sitting for long periods of time and a history of smoking 20 packs/yr; however, he stopped smoking 10 years prior to admission. No similar symptoms were found in his family. The initial assessment revealed cyanosis and decreased right breath sounds. No pitting edema was observed in the lower extremities. The laboratory tests showed the following results: alanine transaminase (ALT): 52 IU/L; aspartate transaminase (AST): 93 IU/L; D-Dimer: >10 μg/mL; N-terminal of the prohormone brain natriuretic peptide (NT-proBNP): 3544 рg/mL; and troponin I: 0.49 ng/mL. The arterial blood gas (ABG) test revealed severe hypoxemia and an oxygenation index of 89 mmHg, and the electrocardiogram showed an S_1_Q_3_T_3_ pattern (Fig. 1). CTPA revealed multiple filling defects in the right main (Fig. 2A), both lobar (Fig. 2B, C) and segmental (Fig. 2D) pulmonary arteries (PAs) and an irregular mass in the right atrium (Fig. 2D). Transthoracic echocardiogram (TTE) showed enlargement of the right chambers and a 54 × 47 mm right atrial mass attached to the top wall with clear margins, an irregular shape, partial rough surface texture, and loose internal structure; the mass moved with the cardiac cycle, and mild prolapse through the leaflets of the tricuspid valve and orifice of inferior vena cava and moderate regurgitation of the tricuspid valves with mild pulmonary hypertension were observed. Compressed venous ultrasonography showed negative results in both lower limbs.

**Figure 3:**
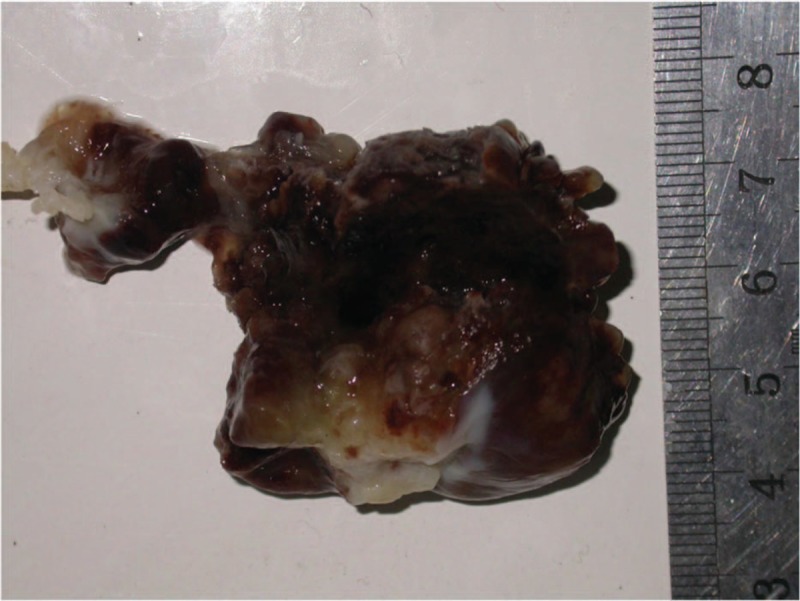
Excised 40 × 50 mm fragile tumor mass with an irregular surface, gelatinous consistency with necrosis and bleeding.

**Figure 4:**
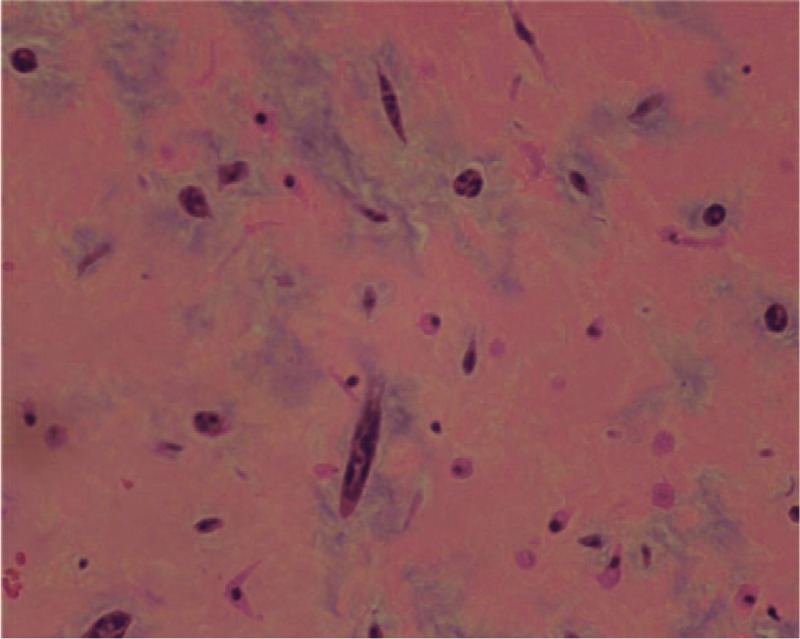
Histology of the excised tumor. The tumor consists of an acid-mucopolysaccharide-rich stroma. Polygonal cells with scant eosinophilic cytoplasm can be observed in the matrix. Hematoxylin–eosin stain.

The surgical approach was a medial sternotomy under extracorporeal circulation. The right atrial wall was opened, and a fragile tumor with a gelatinous consistency and necrosis that measured 40 × 50 mm and adhered to the interatrial septum (Fig. 3) and a 30 × 20 × 70 mm tumor embolus in the right main PA, with the distal end near the right upper PA, were observed. The tumor cells expressed CD34 and calretinin and were negative for CK and SMA. The histopathological examination confirmed a myxoma (Fig. 4) in the right atrium and right pulmonary artery. The patient followed an uneventful course during the 6-year follow-up.

## 3 Discussion

Cardiac tumors are rare, and most of these tumors originate from metastasis. The incidence of primary cardiac tumors (PCTs) in autopsy ranges from 0.02% to 2.8%. In total, 30% to 50% of PCTs are myxomas, with 75% occurring in the left atrium and only 10% to 20% arising in the right atrium; myxomas may develop from the embryonic or primitive gut remnants.^[5–7]^ Histologically, myxomas consist of an acid-mucopolysaccharide rich stroma. Polygonal cells arranged in a single layer or small clusters are scattered among the matrix.

Right atrial myxoma (RAMs) may remain asymptomatic or appear with constitutional, obstructive, or embolic symptoms according to the size, fragility, mobility, and location of the tumor as well as body position and activity.^[5,8]^ The nonspecific constitutional signs, which are present in 10% to 45% of patients with myxomas, are fatigue, fever, dyspnea, chronic anemia, weight loss, general arthralgia, and elevated interleukin-6, erythrocyte sedimentation rate, and c-reaction protein levels.^[8]^ Therefore, the results of the laboratory tests may mimic those for rheumatic disorders. These signs are more common for patients with large, multiple, or recurrent tumors and usually return to normal after resection.^[9]^ Pulmonary embolisms of the RAM fragments or thrombi from the surface may also occur, resulting in dyspnea, pleuritic chest pain, hemoptysis, syncope, pulmonary hypertension, right heart failure, or even sudden death. Acute abdominal pain was mentioned in 2 cases.^[10]^ Embolic events are common in patients with cardiac myxomas, with an incidence ranging from 30% to 40%.^[5]^ Our patient presented with a pulmonary embolism as the initial manifestation.

In cases of RAMs with pulmonary tumoral embolisms, a small size, villous or irregular surface and presence of multiple foci are the most common risk factors associated with embolization.^[11]^ The surface of the RAM was irregular in our case. In the literature, the duration period ranged from 1 day to 3.5 years. The age of the patients ranged from 17 to 76 years (mean age 42.8 years), with a higher incidence in women (20/35, 57%) than in men. In these cases, the RAMs were usually attached by a short pedicle to the interatrial septum (22/35), mostly in the fossa ovalis, while the others were in the free wall, crista terminalis, and Koch triangle or had multiple origins. Most of the patients were diagnosed with TTE, computed tomography (CT), transesophageal echocardiography (TEE), or magnetic resonance imaging (MRI), while the other patients were diagnosed with angiography and autopsy. In almost all cases, the treatment was surgery to remove the intra-atrial myxomas and the pulmonary emboli, which were usually tumoral (Table 1). The majority of such patients recovered well after surgery. Four preoperative deaths and 2 postoperative deaths were reported. Right atrial thrombosis, transient ischemic attack (TIA), ischemic hepatitis, and renal failure were rare complications. In our case, the surface of the RAM originated from the right atrial fossa ovalis and was irregular. We confirmed the diagnosis by TTE and CT, as in most of the published cases.

**Table 1:**
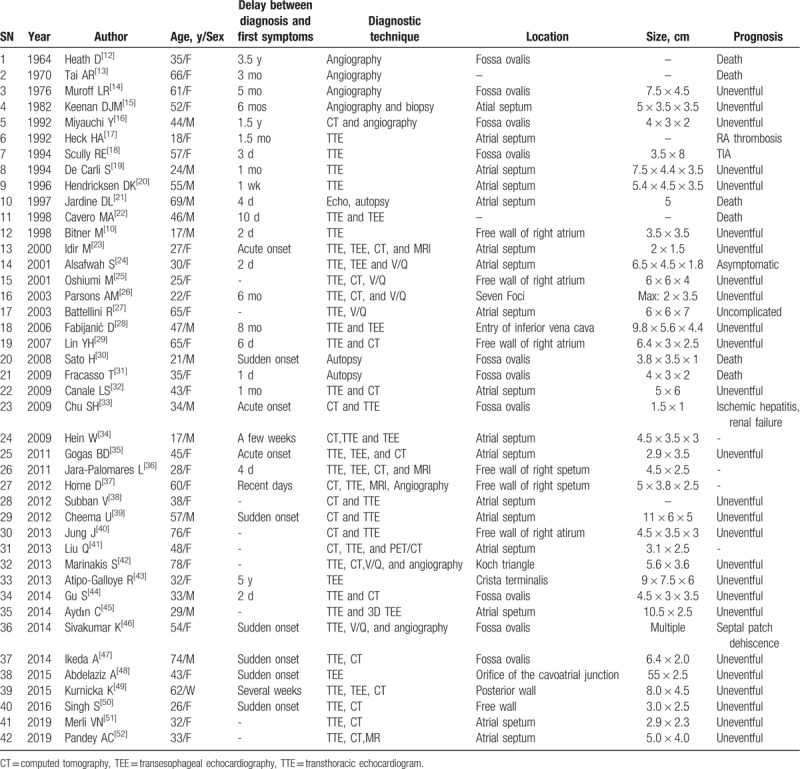
Right atrial myxomas complicated with pulmonary embolism in the literature.

TTE and TEE are the most commonly used diagnostic methods in the detection and initial description of atrial myxomas.^[23]^ TTE has a sensitivity of nearly 95% for confirming cardiac myxomas, and the sensitivity of TEE is nearly 100%.^[45]^ TTE can facilitate bedside testing to safely detect myxomas in fatal pulmonary embolisms, as in our patient. TEE produces clear images of small tumors (1–3 mm in diameter), especially in overweight patients with poor TTE images.^[53]^ Compared with TTE, TEE also permits a clearer picture of the attachment of the tumor and more precise characterization of the size, shape, surface, inner structure, and location of the mass.^[54]^ Although TEE is a semi-invasive diagnostic test with a very low rate of significant complications, lethal pulmonary embolisms during the TEE procedure have been reported.^[22]^

Compared with echocardiography, multidetector computed tomography (MSCT) and cardiac magnetic resonance imaging (CMR) are more accurate in determining the relationship of the myxoma to normal intracardiac structures, tumor infiltration into the pericardium, extension to adjacent vasculature and mediastinal structures, and presence of pulmonary arteries emboli and aiding in surgical planning.^[55,56]^ RAMs manifest as a low-attenuation intra-atrial masses with a smooth, irregular or villous surface on MSCT. Calcifications are seen in approximately 14% of the cases and are more common in right-sided lesions than in left-sided lesions. Contrast enhancement is usually not apparent in the arterial phase, but heterogeneous enhancement has been reported in studies performed with a longer time delay.^[57,58]^ Varying amounts of myxoid, calcified, hemorrhagic, and necrotic tissue give myxomas a heterogeneous appearance on T1- and T2-weighted images. Delayed enhancement is typical and usually patchy in nature. Steady-state free precession (SSFP) sequences may slow prolapse through the tricuspid valve in the diastole phase and may reveal the attachment points of stalk lesions. Reconstruction of cine gradient-recalled echo (GRE) images enables the assessment of lesion mobility and attachment.^[59]^

The imaging technique of positron emission tomography with 2-deoxy-2-[18F] fluoro-D-glucose and CT (^18^F-FDG PET/CT) can help noninvasively confirm the malignancy before the surgery.^[41]^ The mean SUV_max_ was 2.8 ± 0.6 in benign cardiac tumors and was significantly higher than this value in both primary and secondary cases of malignancy (8.0 ± 2.1 and 10.8 ± 4.9, respectively). The SUV_max_ measurements of myxomas range from 1.6 to 4. Malignancies were determined with a sensitivity of 100% and specificity of 86% with a cut-off SUV_max_ value of 3.5. A weak correlation between the SUV_max_ and the size of the tumors was found due to the partial volume effect, cardiac motion, and respiratory movement.^[60]^ Angiography is an invasive investigation that presents an additional risk of inducing tumor migration and is only suitable for suspected acute coronary heart diseases.^[37]^

Surgical removal of the RAM with pulmonary embolisms is the treatment of choice and is usually curative.^[44,45]^ The crucial aspects of surgery are the measurements for bicaval cannulation to prevent intraoperative embolisms,^[27]^ en bloc excision of the myxoma with a wide cuff of normal tissue, removal of the fragments in the pulmonary arteries, and use of moderate or deep hypothermia, low circulatory flow or total circulatory arrest based on the extent and sites of the emboli.^[44]^ Surgical treatment leads to complete resolution with low rates of recurrence and good long-term survival.

The overall recurrence rate is approximately 1% to 3% for sporadic atrial myxomas,^[5,61]^ which grow an average of 0.24 to 1.6 cm per year. The risk for recurrent pulmonary embolisms after resection has been reported to be 0.4% to 5.0%, and the interval from excision to recurrence has been reported to range from a few months to 8 years.^[62]^ The reasons for RAM recurrence include a multifocal origin, incomplete surgical resection, familial disposition, or abnormal DNA ploidy patterns. For long-term observations, postoperative annual TTE and V/Q (ventilation perfusion scan) scans should be performed to detect the eventual recurrence of new myxomas and pulmonary embolisms. Excision of the recurrent lesions may be the only treatment choice because of the poor effects of chemotherapy and radiation.^[28]^

In conclusion, RAMs should be considered unlikely reasons for fatal pulmonary embolisms. The main treatment is excision of the masses from the atrium and pulmonary arteries. Annual TTE and CT imaging are suggested for a period of 8 years, which represents the period that the risk of recurrence has been reported.

## Author contributions

**Data curation:** Guofeng Ma, Yong Zhou.

**Resources:** Guofeng Ma, Yongtao He, Dan Wang, Yong Zhou.

**Supervision:** Kejing Ying.

**Writing – original draft:** Guofeng Ma.

**Writing – review & editing:** Ruifeng Zhang.
